# The Relation of Ambulatory Heart Rate with All-Cause Mortality among Middle-Aged Men: A Prospective Cohort Study

**DOI:** 10.1371/journal.pone.0121729

**Published:** 2015-03-26

**Authors:** Mette Korshøj, Mark Lidegaard, France Kittel, Koen Van Herck, Guy De Backer, Dirk De Bacquer, Andreas Holtermann, Els Clays

**Affiliations:** 1 National Research Centre for the Working Environment, Copenhagen, Denmark; 2 School of Public Health, Université Libre de Bruxelles, Brussels, Belgium; 3 Department of Public Health, Ghent University, Ghent, Belgium; University of Tampere, FINLAND

## Abstract

The aim of this study was to investigate the association between average 24-hour ambulatory heart rate and all-cause mortality, while adjusting for resting clinical heart rate, cardiorespiratory fitness, occupational and leisure time physical activity as well as classical risk factors. A group of 439 middle-aged male workers free of baseline coronary heart disease from the Belgian Physical Fitness Study was included in the analysis. Data were collected by questionnaires and clinical examinations from 1976 to 1978. All-cause mortality was collected from the national mortality registration with a mean follow-up period of 16.5 years, with a total of 48 events. After adjustment for all before mentioned confounders in a Cox proportional hazards regression analysis, a significant increased risk for all-cause mortality was found among the tertile of workers with highest average ambulatory heart rate compared to the tertile with lowest ambulatory heart rate (Hazard ratio = 3.21, 95% confidence interval: 1.22–8.44). No significant independent association was found between resting clinic heart rate and all-cause mortality. The study indicates that average 24-hour ambulatory heart rate is a strong predictor of all-cause mortality independent from resting clinic heart rate, cardiorespiratory fitness, occupational and leisure time physical activity and other classical risk factors among healthy middle-aged workers.

## Introduction

Resting heart rate measured at the clinic is shown to be a strong predictor of cardiovascular [1–2] and all-cause mortality [3–8]. Because it is a rather quick and easy measure, it has recently received considerable attention as a mean to stratify risk for hypertension, cardiovascular disease and all-cause mortality. The role of resting heart rate is therefore emphasized in the European guidelines for treating hypertension and cardiovascular disease [9].

The strain on the cardiovascular system is generally acknowledged to be better investigated by repeated ambulatory measurements throughout daily living than during standardized rest in an artificial and often more stressful environment, as in the clinic [7, 10–11]. Moreover, ambulatory heart rate, continuously measured during a day, is shown to have a better reproducibility than resting clinical heart rate [12]. Therefore, ambulatory heart rate should theoretically be a stronger predictor for cardiovascular and all-cause mortality than resting clinic heart rate. However, studies investigating the predictive role of ambulatory heart rate for cardiovascular and all-cause mortality have found contrasting results [13–15].

The contrasting results may be caused by lacking adjustment for potential confounding factors like cardiorespiratory fitness and occupational and leisure time physical activity, previously shown to be related to ambulatory heart rate [11, 16] and mortality [17–20]. Another explanation of the conflicting results may be that most of the existing studies on ambulatory heart rate are not based on continuous measurements of heart rate throughout a day, but on relatively few measurements over short periods throughout the day [3, 13, 15]. Moreover, we are not aware of previous studies investigating if average ambulatory heart rate is associated with all-cause mortality, independent from resting clinical heart rate. Therefore, the aim of the study was to investigate the association between 24-hours continuously measured ambulatory heart rate and all-cause mortality adjusted for resting clinic heart rate, cardiorespiratory fitness, occupational and leisure time physical activity as well as classical risk factors.

## Materials and Methods

### Study design and population

The Belgian Physical Fitness Study (BELFIT) was a prospective cohort study among 2,363 male workers, the study protocol and the main results were described previously [21]. The baseline study was conducted in 1976–1978, the data collection included standardized examinations of bio-clinical measurements, a submaximal graded exercise test and various questionnaires; the response rate was 75%. Data collection was carried out by trained researchers at the Occupational Medicine Departments of the participating companies. At the enrolled companies all men who were regularly employed and aged 40–55 years old were invited to participate. Ambulatory ECG recordings were obtained in only a subsample of participants, but this selection was based on a choice of factories where it was feasible to fit all workers with an ambulatory ECG monitor for a 24-h period. An informed consent was obtained from all participants before inclusion in the study. The study was approved by the ethics committees of Ghent University and the Free University of Brussels.

**Fig 1:**
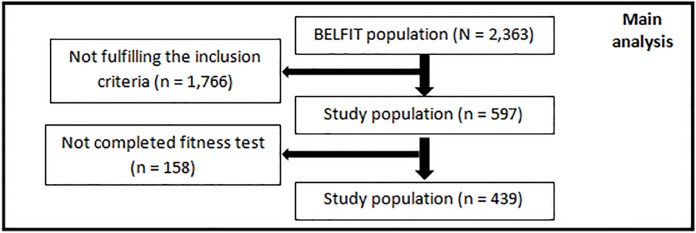
Flow chart of the study population. The complete BELFIT population included 2,363 men. Inclusion criteria for the present study (availability of resting heart rate and diurnal heart rate data from ECG recordings, no history of coronary heart disease and no medical treatment for coronary heart disease or arterial hypertension) reduced the number to 597 participants, with 439 men having completed the exercise test on a bicycle ergometer.

Inclusion in the present analysis was based on the following criteria: no history of coronary heart disease, not medically treated for coronary heart disease or arterial hypertension and availability of data on resting heart rate and mean diurnal heart rate from the ECG recordings. An additional inclusion criterion for the analysis was completion of the cardiorespiratory fitness test. This selection resulted in a final study population of 439 individuals (Fig. 1).

### Questionnaire data

Several self-administered questionnaires were completed by the participants, including information about age, smoking behavior and education. Those stating to regularly smoke were defined as current smokers. Primary school was classified as a low educational level, secondary school as a medium educational level, and high school or university as a high educational level. Two additional questionnaires assessing occupational physical activity and leisure time physical activity were administered by the interviewer. Level of leisure time physical activity (LTPA) was collected by the validated Minnesota leisure time physical activity questionnaire [22], estimating the individual participants’ mean energy expenditure during leisure hours. Activity metabolic index was calculated in three categories with following intensity codes; light activity metabolic index of 2.0–4.0, moderate activity metabolic index of 4.5–5.5 and heavy activity metabolic index of ≥ 6.0. Additionally total activity metabolic index was calculated. The calculated activity metabolic index expresses the accumulated energy expenditure in kcal during the previous three months. For the current analysis, we used LTPA of heavy intensity during the past 3 months as the exposure variable, in line with international guidelines regarding the health impact of physical activity [23]. Occupational physical activity (OPA) was estimated by detailed information concerning attitudes, postures and movements on a regular working day and derived caloric expenditure values for various activities in occupation. Thereby the occupational energy expenditure was calculated in kcal/hour, including a fixed basal metabolism on 73 kcal/hour [21]. Both LTPA and OPA were divided into tertiles, defined as low, medium and high level of physical activity.

### Ambulatory ECG monitoring

Heart rate was collected by 24-hour ambulatory ECG recordings during a regular working day. For measuring continuous ambulatory ECG, bipolar electrodes were placed in the V5-V5R position and connected to a phase-locked loop speed control tape recorder at 2 mm/s (Medilog type I, Oxford Medical). The Syne Tec software (version 2.00 ELA medical, F-Le Plessis-Robinson) was used for digitally sampling and analysis of the ECG recordings. From the ECG recordings the mean heart rate over 24-hour (beats per minute (bpm)) was calculated. Only recordings with >90% of qualiWed sinus beats for at least a 23-hour period were included in the analysis of heart rate. The average duration of the ECG recordings was 23.3 hours.

### Bio-clinical examination

Anthropometrics (height, weight) and cardiovascular risk factors (blood pressure, total cholesterol and electrocardiographic recording) were collected following standardized measurement protocols [21]. Body mass index (BMI) was calculated by dividing body weight (kg) by the square of the height (m). Total cholesterol was centrally measured in a laboratory.

### Exercise test

To estimate physical working capacity a submaximal graded exercise test was performed on a bicycle ergometer [21]. The initial workload was fixed at 75 watts, every 2.5 minutes 25 watts increments were put on. The target intensity was 80% of the predicted maximal heart rate, in the study population this corresponds to a heart rate of 150 bpm. The cardiorespiratory fitness was calculated by interpolation, by the definition of a workload expressed in watts at a heart rate of 150 bpm. Exclusion criteria for cardiorespiratory fitness testing were based on medical history, resting blood pressure (≥170/105 mmHg), and resting electrocardiograms following the Minnesota Code readings [21].

### Mortality follow-up

Mortality follow-up was conducted from vital status based on information from the National Registry of the Belgian population. All events of mortality were linked to the National Institute for Statistics for the coded death certificates [24]. The mean mortality follow-up time was 16.5 years (SD 3.3) and median 17.7 years (IQR 17.5–18.1).

### Statistical analysis

Descriptive statistics are presented by numbers and percentages, mean values and standard deviation (SD), or median values and inter-quartile range (IQR). Tertile groups of heart rate (resting or ambulatory) were compared by means of analysis of variance F-test in normally distributed continuous variables, Kruskal-Wallis test in skewed continuous variables, or Chi²-test in categorical variables.

The following potential confounding variables were treated as continuous variables: age, BMI, systolic blood pressure, total cholesterol and cardiorespiratory fitness. Since systolic and diastolic blood pressure were highly correlated (Pearson r = 0.67), only systolic blood pressure was included in the model as a confounder. The following potential confounding variables were treated as categorical variables: education, heavy LTPA during the past 3 months and OPA.

Cox proportional hazard ratio was used to assess the prospective association between average heart rate and time to all-cause mortality. Average diurnal and resting heart rate were treated in the statistical models as both continuous variables and as categorical variables (tertiles). Covariates were stepwise included with forced-entry in the models. The first model included age, the second model included age, education, BMI, LTPA, smoking, systolic blood pressure, total cholesterol, OPA and cardiorespiratory fitness, and the third model was additionally mutually adjusted for resting/ambulatory heart rate. In the sample of 597 men in whom no complete data on cardiorespiratory fitness were available, an additional sensitivity analysis was conducted repeating the fully adjusted Cox regression analysis with the exception of cardiorespiratory fitness as confounder. A p-value of 0.05 was considered statistically significant. All analyses were conducted in IBM SPSS Statistics version 20.

## Results

During a mean follow-up time of 16.5 years, 48 participants (10.9%) died from all-causes. Baseline characteristics of the study population are shown in Table 1. Compared with the entire BELFIT population, the sub-population of 439 performing the fitness test was significantly younger, included fewer smokers and had a lower BMI and systolic/diastolic blood pressure, however no significant differences in terms of resting heart rate or mortality were observed.

**Table 1:** Description of baseline characteristics in 439 men.

	**% (N) or mean (SD) or median (IQR)**
Age in years: mean (SD)	46.4 (4.2)
Educational level: % (N)	
Low	22.5 (98)
Medium	65.5 (285)
High	12.0 (52)
Occupational class: % (N)	
White-collar	75.2 (330)
Blue-collar	24.8 (109)
Current smoker: % (N)	41.9 (184)
Body mass index (kg/m²): mean (SD)	25.3 (2.7)
Weight group: % (N)	
Normal weight (BMI<25kg/m²)	45.8 (201)
Overweight (25kg/m²≤BMI<30kg/m²)	49.9 (219)
Obesity (BMI≥30kg/m²)	4.3 (19)
Systolic blood pressure (mmHg): mean (SD)	132.2 (13.5)
Diastolic blood pressure (mmHg): mean (SD)	81.9 (10.6)
Total cholesterol (mg/dl): mean (SD)	234.7 (33.6)
HDL cholesterol (mg/dl): mean (SD)	54.7 (14.3)
Cardiorespiratory fitness (watts/kg): mean (SD)	1.46 (0.27)
Occupational physical activity: median kcal/working hour (IQR)	1,567 (1,221–2,184)
Groups of occupational physical activity: median kcal/working hour (IQR)	
Low (n = 147; 33.5%)	1,114 (1,023–1,222)
Medium (n = 145; 33.0%)	1,567 (1,435–1,814)
High (n = 147; 33.5%)	2,484 (2,184–2,940)
Heavy leisure time physical activity: median total kcal in last 3 months (IQR)	1,080 (0–5,280)
Groups of heavy leisure time physical activity: median total kcal in last 3 months (IQR)	
Low (n = 183; 41.7%)	0 (0–0)
Medium (n = 123; 28.0%)	1,584 (1,056–2,560)
High (n = 133; 30.3%)	7,920 (5,760–13,524)
Resting clinical heart rate: mean beats/min (SD)	69.7 (11.1)
Groups of resting clinical heart rate: mean beats/min (SD)	
Low (n = 141; 32.1%)	58.1 (4.4)
Medium (n = 152; 34.6%)	68.5 (2.7)
High (n = 146; 33.3%)	82.1 (7.7)
Ambulatory 24-hour heart rate: mean beats/min (SD)	81.8 (8.9)
Groups of ambulatory 24-hour heart rate: mean beats/min (SD)	
Low (n = 146; 33.3%)	72.3 (4.8)
Medium (n = 146; 33.3%)	81.8 (2.0)
High (n = 147; 33.5%)	91.1 (5.6)

SD, standard deviation; BMI, body mass index; IQR, inter-quartile range.

Significant differences between the tertile groups of average ambulatory heart rate were found for smoking and cardiorespiratory fitness. Participants with high average ambulatory heart rate generally had a higher proportion of smokers and a lower cardiorespiratory fitness than participants with low ambulatory heart rate.

Significant differences between the tertile groups of resting clinic heart rate were found for systolic blood pressure, OPA and cardiorespiratory fitness. Participants with high resting clinic heart rate generally had a higher systolic blood pressure, a lower OPA and a lower cardiorespiratory fitness compared with participants with a low resting clinic heart rate.

Table 2 shows the associations of both continuous and categorical (in tertiles) resting clinical and average ambulatory diurnal heart rate with all-cause mortality. The continuous measure of resting heart rate and average ambulatory diurnal heart rate was significantly related to all-cause mortality in the first model adjusted for age: each increase of 1 bpm in heart rate was associated with an elevated risk for all-cause mortality by 4% and 6% (95%CI: 1.02–1.07; 1.03–1.09) for resting clinical heart rate and ambulatory diurnal heart rate, respectively. In the fully adjusted model, tendencies for significant associations were found for both resting heart rate and average ambulatory diurnal heart rate.

**Table 2:** Associations between continuous and categorized resting clinical and average continuously measured 24-hour ambulatory heart rate and all-cause mortality in 439 men.

	**All-cause mortality**				
	**N of events (%)**	***P* chi²**	**HR** ^**a**^ **(95% CI)**	**HR** ^**b**^ **(95% CI)**	**HR** ^**c**^ **(95% CI)**
Ambulatory Heart Rate in tertiles		<0.001			
Low	7 (4.8)		1	1	1
Medium	14 (9.6)		2.22 (0.89–5.50)	1.98 (0.78–5.04)	1.94 (0.74–5.10)
High	27 (18.4)		**4.57 (1.98–10.51)**	**3.46 (1.39–8.61)**	**3.21 (1.22–8.44)**
Ambulatory Heart Rate (continuous per single beat)	-	-	**1.06 (1.03–1.09)**	1.03 (1.00–1.07)	1.02 (0.99–1.06)
Ambulatory Heart Rate (continuous per 10 beats)	-	-	**1.77 (1.32–2.37)**	1.39 (1.00–1.94)	1.27 (0.88–1.84)
Resting Heart Rate in tertiles		<0.01			
Low	9 (6.4)		1	1	1
Medium	13 (8.6)		1.24 (0.53–2.91)	1.09 (0.45–2.65)	0.87 (0.35–2.17)
High	26 (17.8)		**3.00 (1.40–6.41)**	1.94 (0.78–4.84)	1.26 (0.48–3.31)
Resting Clinical Heart Rate (continuous per single beat)	-	-	**1.04 (1.02–1.07)**	1.03 (1.00–1.06)	1.02 (0.99–1.06)
Resting Clinical Heart Rate (continuous per 10 beats)	-	-	**1.49 (1.18–1.89)**	1.34 (0.98–1.83)	1.22 (0.86–1.74)

Significant results at the 0.05 level are in bold

^a^ Cox proportional hazards regression analysis: adjusted for age

^b^ Cox proportional hazards regression analysis: adjusted for age, education, body mass index, leisure time physical activity, smoking, systolic blood pressure, total cholesterol, occupational physical activity and cardiorespiratory fitness

^c^ Cox proportional hazards regression analysis: adjusted for age, education, body mass index, leisure time physical activity, smoking, systolic blood pressure, total cholesterol, occupational physical activity, cardiorespiratory fitness and resting clinical heart Rate/ diurnal heart rate, respectively.

Based on categorized measures of average ambulatory diurnal heart rate, those with a high average heart rate had an increased risk for all-cause mortality in the fully adjusted model (HR:3.21, 95%CI: 1.22–8.44) compared to those with a low average ambulatory heart rate.

For resting clinic heart rate, a significantly increased risk for all-cause mortality was found for the tertile with highest resting clinical heart rate compared with the lowest in the age-adjusted model (HR:3.00, 95%CI: 1.40–6.41). However, the association did not remain significant and materially reduced the hazard ratio in the fully adjusted model (HR: 1.26, 95%CI: 0.48–3.31).

In the sample of 597 men without complete data on cardiorespiratory fitness, we repeated the fully adjusted Cox regression analysis (model c from Table 2) with the exception of cardiorespiratory fitness as confounder. This additional sensitivity analysis yielded very similar conclusions about the independent relations of resting vs. ambulatory heart rate with total mortality. Hazard rates for ambulatory heart rate were 1.63 (95% CI: 0.79–3.36) for the medium category and 2.45 (95% CI: 1.19–5.05) for the highest tertile group. No significant independent associations were observed for resting heart rate, with hazard rates in the fully adjusted model being 1.12 (95% CI: 0.58–2.15) for the medium group and 1.32 (95% CI: 0.69–2.53) for the highest tertile group.

## Discussion

In this study conducted within a group of male workers free of coronary heart disease at baseline, average continuously measured ambulatory heart rate was shown to be significantly associated with all-cause mortality while adjusting for resting clinical heart rate, cardiorespiratory fitness, OPA and LTPA, as well as for classical risk factors. After adjusting for all these potential confounders, those with high average ambulatory heart rate had more than three times increased risk for all-cause mortality compared to those with low average ambulatory heart rate. Those with medium ambulatory heart rate did not have a significantly increased risk for all-cause mortality, although the effect sizes indicate a dose-response relationship between ambulatory heart rate and all-cause mortality. In the fully adjusted model we mutually adjusted for resting vs. diurnal heart rate, in order to investigate whether or not both measures were related to mortality over and above each other. Resting clinic heart rate did not significantly predict all-cause mortality independent from cardiorespiratory fitness, OPA and LTPA, average ambulatory heart rate as well as classical risk factors. These findings support that average heart rate throughout the day, more than resting heart rate, is a strong independent predictor for all-cause mortality in healthy middle-aged working men.

Previous studies have shown that resting clinic heart rate is a relatively strong predictor for all-cause mortality [3–8]. Moreover, it was shown that the significant association between resting clinic heart rate and all-cause mortality remains after adjustments for ambulatory heart rate [7, 13–14]. In the Copenhagen Male Study, elevated resting heart rate was a risk factor for mortality independent of physical fitness and LTPA [8]. However, we are not aware of previous studies on the association between resting clinic heart rate and mortality also adjusting simultaneously for cardiorespiratory fitness, OPA, LTPA, average ambulatory heart rate, and classical risk factors.

Previous studies investigating the predictive role of ambulatory heart rate for cardiovascular and all-cause mortality have found contrasting results, observing both significant positive associations and some zero findings [13–15]. This may be due to lacking or insufficient adjustments of relatively strong potential confounders in the association between heart rate and mortality like cardiorespiratory fitness, physical activity and resting clinic heart rate [13–15]. Another explanation may be that different measures of ambulatory heart rates are applied [13–15], or that the previous studies have used measurement of ambulatory heart rate with relatively few measurements over short periods throughout the day [3, 13, 15] instead of continuous measurements of heart rate throughout a day like in this study.

One potential physiological mechanism for the relation between average ambulatory heart rate throughout a day and all-cause mortality is the association between heart rate and blood pressure. An elevated heart rate will shorten time spent in systole and prolong time spent in diastole, leading to a longer period with greater turbulence and less laminar blood flow in the lumen, which leads to suboptimal shear stress [25–26]. Suboptimal shear stress can potentially initiate endothelial damage in the arteries, absorption of lipids in the arterial wall, and finally inflammation which may lead to arteriosclerosis [27]. Other possible underlying mechanisms that could account for the observed association are insulin resistance, systemic low-grade inflammation and increased sympathetic activation, factors which are known to increase the risk for cancer mortality [28].

The findings of this study suggest that average ambulatory heart rate is an independent predictor—over and above resting clinic heart rate—of premature mortality when adjusting for potential confounders including measures of fitness and physical activity. Because average ambulatory heart rate is easy to measure and analyze with relatively inexpensive monitors, it seems to stand out as an appropriate clinical measure of cardiovascular strain.

### Strengths and limitations

Strengths of this study are the ambulatory measurement of heart rate throughout a day, adjustment for OPA, LTPA and cardiorespiratory fitness, as well as the long-term registration of complete all-cause mortality following rigorous follow-up procedures. Moreover, the ambulatory heart rate was measured by ambulatory ECG recordings, making beat by beat analysis of heart rate throughout the entire measurement period available. Additionally, this study population was free from history of cardiovascular disease.

A limitation of the study is the insufficient sample size and follow-up time for investigating less incident outcomes like cardiovascular mortality. Another limitation is the lacking information about working time, leisure time and sleep time throughout the day, not permitting time/setting specific analyses between heart rate and all-cause mortality. Additionally, there could be a bias from the healthy worker selection, increased by the inclusion criteria of a completed fitness test. The population is exclusively male and thus information on these associations among women is lacking.

## Conclusion

The present study indicates that average 24-hour continuously measured ambulatory heart rate is a strong predictor of all-cause mortality independent from resting clinic heart rate, cardiorespiratory fitness, OPA and LTPA, and other classical risk factors in healthy middle-aged working men.

## References

[1] Greenland P, Daviglus ML, Dyer AR, Liu K, Hunag CF, Goldberger JJ, et al Resting heart rate is a risk factor for cardiovascular and noncardiovascular mortality: the Chicago Heart Association Detection Project in Industry. Am J Epidemiol. 1999;149(9): 853–862. 10221322 10.1093/oxfordjournals.aje.a009901

[2] Kannel WB, Belanger A, D’Agostino R, Israel I. Physical activity and physical demand on the job and risk of cardiovascular disease and death: The Framingham Study. Am Heart J. 1986;112(4): 820–825. 3766383 10.1016/0002-8703(86)90480-1

[3] Ben-Dov IZ, Kark JD, Ben-Ishay D, Mekler J, Ben-Arie L, Bursztyn M. Blunted Heart Rate Dip During Sleep and All-Cause Mortality. Arch Intern Med. 2007;167(19): 2116–2121. 17954807 10.1001/archinte.167.19.2116

[4] Cooney MT, Vartiainen E, Laakitainen T, Juolevi A, Dudina A, Graham IM. Elevated resting heart rate is an independent risk factor for cardiovascular disease in healthy men and women. Am Heart J. 2010;159(4): 612–619. 10.1016/j.ahj.2009.12.029 20362720

[5] Fox K, Borer JS, Camm AJ, Danchin N, Ferrari R, Lopez Sendon JL, et al Resting Heart Rate in Cardiovascular Disease. J Am Coll Cardiol. 2007;50(9): 823–830. 17719466 10.1016/j.jacc.2007.04.079

[6] Jensen MT, Marott JL, Allin KH, Nordestgaard BG, Jensen GB. Resting heart rate is associated with cardiovascular and all-cause mortality after adjusting for inflammatory markers: The Copenhagen City Heart Study. Eur J Cardiovasc Prev Rehabil. 2012;19(1): 102–108. 10.1177/1741826710394274 21525123

[7] Johansen CD, Olsen RH, Pedersen LR, Kumarathurai P, Mouridsen MR, Binici Z, et al Resting, night-time, and 24 h heart rate as markers of cardiovascular risk in middle-aged and elderly men and women with no apparent heart disease. Eur Heart J. 2013;34(23): 1732–1739. 10.1093/eurheartj/ehs449 23306958

[8] Jensen MT, Suadicani P, Ole Hein H, Gyntelberg F. Elevated resting heart rate, physical fitness and all-cause mortality: a 16-year follow-up in the Copenhagen Male Study. Heart. 2013;99: 882–887. 10.1136/heartjnl-2012-303375 23595657 PMC3664385

[9] Graham I, Atar D, Borch-Johnsen K, Boysen G, Burell G, Cifkova R, et al European guidelines on cardiovascular disease prevention in clinical practice: executive summary: Fourth Joint Task Force of the European Society of Cardiology and Other Societies on Cardiovascular Disease Prevention in Clinical Practice (Constituted by representatives of nine societies and by invited experts). Eur Heart J. 2007;28(19): 2375–2414. 17726041 10.1093/eurheartj/ehm316

[10] Hansen TW, Kikuya M, Thijs L, Bjørklund-Bodegård K, Kuznetsova T, Ohkubo T, et al Prognostic superiority of daytime ambulatory over conventional blood pressure in four populations: a meta-analysis of 7030 individuals. J Hypertens. 2007;25(8): 1554–1564. 17620947 10.1097/HJH.0b013e3281c49da5

[11] Imai Y, Hozawa A, Ohkubo T, Ohmori K, Kikuya M, Hashimoto J, et al Heart rate measurement and outcome. Blood Press Monit. 2003;8(1): 53–55. 12604938 10.1097/00126097-200302000-00011

[12] Palatini P, Winnicki M, Santonastaso M, De Venuto G, Zanata G, Bertolo O, et al Reproducibility of heart rate measured in the clinic and with 24-hour intermittent recorders. Am J Hypertens. 2000;13(1): 92–98. 10678277 10.1016/s0895-7061(99)00170-3

[13] Hansen TW, Thijs L, Boggia J, Li Y, Kikuya M, Bjørklund-Bodegård K, et al Prognostic Value of Ambulatory Heart Rate Revisited in 6928 Subjects From 6 Populations. Hypertension. 2008;52(2): 229–235. 10.1161/HYPERTENSIONAHA.108.113191 18574073

[14] Hozawa A, Inoue R, Ohkubo T, Kikuya M, Metoki H, Asayama K, et al Predictive value of ambulatory heart rate in the Japanese general population: the Ohasama study. J Hypertens. 2008;26(8): 1571–1576. 10.1097/HJH.0b013e3283041172 18622234

[15] Palatini P, Thijs L, Staessen JA, Fagard RH, Bulpitt CJ, Clement DL, et al Predictive Value of Clinic and Ambulatory Heart Rate for Mortality in Elderly Subjects With Systolic Hypertension. Arch Intern Med. 2002;162(20): 2313–2321. 12418945 10.1001/archinte.162.20.2313

[16] Karvonen MJ, Kentala E, Mustala O. The effects of training on heart rate: a longitudinal study. Ann Med Exp Biol Fenn. 1957;35(3): 307–315. 13470504

[17] Blair SN, Kohl HW 3rd, Paffenbarger RS Jr, Clark DG, Cooper KH, Gibbons LW. Physical Fitness and All-Cause Mortality: A Prospective Study of Healthy Men and Women. JAMA. 1989;262(17): 2395–2401. 2795824 10.1001/jama.262.17.2395

[18] Holtermann A, Marott JL, Gyntelberg F, Søgaard K, Suadicani P, Mortensen OS, et al Does the Benefit on Survival from Leisure Time Physical Activity Depend on Physical Activity at Work? A Prospective Cohort Study. PLoS ONE. 2013;8(1): e54548 10.1371/journal.pone.0054548 23349926 PMC3547911

[19] Paffenbarger RS, Laughlin ME, Gima AS, Black RA. Work Activity of Longshoremen as Related to Death from Coronary Heart Disease and Stroke. N Engl J Med. 1970;282(20): 1109–1114. 5439408 10.1056/NEJM197005142822001

[20] Clays E, Lidegaard M, De Bacquer D, Van Herck K, De Backer G, Kittel F, et al The Combined Relationship of Occupational and Leisure-Time Physical Activity With All-Cause Mortality Among Men, Accounting for Physical Fitness. Am J Epidemiol. 2014;179(5): 559–566. 10.1093/aje/kwt294 24305575

[21] Sobolski J, de Backer G, Degre S, Kornitzer M, Denolin H. Physical Activity, Physical Fitness and Cardiovascular Diseases: Design of a Prospective Epidemiologic Study. Cardiology. 1981;67(1): 38–51. 7459907 10.1159/000173227

[22] Taylor HL, Jacobs DR, Schucker B, Knudsen J, Leon AS, De Backer G. A questionnaire for the assessment of leisure time physical activities. J Chronic Dis. 1978;31(12): 741–755. 748370 10.1016/0021-9681(78)90058-9

[23] Haskell WL, Lee I-M, Pate RR, Powell KE, Blair SN, Franklin BA, et al Physical Activity and Public Health: Updated Recommendation for Adults from the American College of Sports Medicine and the American Heart Association. Med Sci Sports Exerc. 2007;39(8): 1423–1434. 17762377 10.1249/mss.0b013e3180616b27

[24] Kittel F, de Smet P, Leynen F, De Backer G, Kornitzer M. Socio-professional level and long-term mortality in three Belgian large-scale studies. Arch Public Health. 2003;61: 3–14.

[25] Glagov S, Zarins C, Giddens DP, Ku DN. Hemodynamics and atherosclerosis. Insights and perspectives gained from studies of human arteries. Arch Pathol Lab Med. 1988;112(10): 1018–1031. 3052352

[26] Palatini P. Heart Rate as an Independent Risk Factor for Cardiovascular Disease: Current Evidence and Basic Mechanisms. Drugs. 2007;67(Supplement 2): 3–13. 10.2165/00003495-200767002-00002 17999559

[27] Sukhova GK, Schönbeck U, Rabkin E, Schoen FJ, Poole AR, Billinghurst RC, et al Evidence for increased collagenolysis by interstitial collagenases-1 and -3 in vulnerable human atheromatous plaques. Circulation. 1999;99(19): 2503–2509. 10330380 10.1161/01.cir.99.19.2503

[28] Jouven X, Escolano S, Celermajer D, Empana J, Bingham A, Hermine O, et al Heart Rate and Risk of Cancer Death in Healthy Men. PLoS ONE. 2011;6(8): e21310 10.1371/journal.pone.0021310 21826196 PMC3149594

